# Prevalence and Associations of Co-occurrence of *NFE2L2* Mutations and Chromosome 3q26 Amplification in Lung Cancer

**DOI:** 10.1055/s-0044-1786004

**Published:** 2024-04-15

**Authors:** Jinfeng Liu, Sijie Liu, Dan Li, Hongbin Li, Fan Zhang

**Affiliations:** 1 Department of Thoracic Surgery, The First Hospital of Hebei Medical University, Shijiazhuang, China; 2 Department of Thoracic Surgery, Beijing Aerospace General Hospital, Beijing, China; 3 Department of General Surgery, Jingxing County Hospital of Hebei Province, Shijiazhuang, China; 4 Department of Oncology, Rongcheng County People's Hospital, Baoding, China; 5 Department of Thoracic Surgery, National Cancer Center/National Clinical Research Center for Cancer/Cancer Hospital, Chinese Academy of Medical Science and Peking Union Medical College, Beijing, China

**Keywords:** *NFE2L2*, lung cancer, 3q36 amplification, co-occurrence mutations, next-generation sequencing

## Abstract

**Background** *NFE2L2* (nuclear factor erythroid-2-related factor-2) encodes a basic leucine zipper (bZIP) transcription factor and exhibits variations in various tumor types, including lung cancer. In this study, we comprehensively investigated the impact of simultaneous mutations on the survival of *NFE2L2* -mutant lung cancer patients within specific subgroups.

**Methods** A cohort of 1,103 lung cancer patients was analyzed using hybridization capture-based next-generation sequencing.

**Results** The *NFE2L2* gene had alterations in 3.0% (33/1,103) of lung cancer samples, including 1.5% (15/992) in adenocarcinoma and 16.2% (18/111) in squamous cell carcinoma. Thirty-four variations were found, mainly in exons 2 (27/34). New variations in exon 2 (p.D21H, p.V36_E45del, p.F37_E45del, p.R42P, p.E67Q, and p.L76_E78delinsQ) were identified. Some patients had copy number amplifications. Co-occurrence with *TP53* (84.8%), *CDKN2A* (33.3%), *KMT2B* (33.3%), *LRP1B* (33.3%), and *PIK3CA* (27.3%) mutations was common. Variations of *NFE2L2* displayed the tightest co-occurrence with *IRF2* , *TERC* , *ATR* , *ZMAT3* , and *SOX2* ( *p* < 0.001). In The Cancer Genome Atlas Pulmonary Squamous Carcinoma project, patients with *NFE2L2* variations and 3q26 amplification had longer median survival (63.59 vs. 32.04 months, *p* = 0.0459) and better overall survival.

**Conclusions** *NFE2L2* mutations display notable heterogeneity in lung cancer. The coexistence of *NFE2L2* mutations and 3q26 amplification warrants in-depth exploration of their potential clinical implications and treatment approaches for affected patients.

## Introduction

*NFE2L2* (nuclear factor erythroid-2-related factor-2), encoding NRF2, is a crucial transcription factor responsible for regulating the expression of genes involved in various cellular processes, such as the antioxidant metabolism, lipid and iron catabolism, and proteostasis. 1 The Cancer Genome Atlas (TCGA) data reveal that *NFE2L2* mutations occur in approximately 20% of squamous cell carcinoma (SCC). 2 Activation of the NRF2 pathway can provide a significant advantage in shielding tumor cells from the detrimental effects of oxidative stress. 3 Cancer cells that exhibit sustained activation of NRF2 often acquire a reliance on this pathway and contribute to the development of malignant phenotypes, ultimately leading to unfavorable prognoses in cancer patients. 4 Accumulating evidence indicates the functional involvement of NRF2 in the progression and development of nonsmall cell lung carcinoma (NSCLC). 5 6 Satoh et al demonstrated a relative decrease in the number of tumors with more malignant characteristics in NRF2 knockout mice, underscoring the importance of NRF2 in the initiation and progression of lung cancer. 7 Furthermore, NRF2 activation has been associated with poor treatment response and prognosis in clinical patients. 8 The unfavorable prognosis observed in *NFE2L2* -mutant NSCLC has been partially attributed to the inadequate response to radiotherapy 9 as well as second- and third-line chemotherapy. 10 This diminished responsiveness is primarily associated with resistance mediated by the mutant Nrf2 pathway. 11 Through bioinformatics analysis of NRF2 transcripts in NSCLC cells, a recurring loss of exon 2 or exons 2/3 in NRF2 mRNA was observed as a result of alternative splicing. 12 The deletion of exon 2 presents a sophisticated mechanism for tumors to enhance NRF2 stability by eliminating its interaction sites—specifically, the DLG and ETGE motifs—with KEAP1. Additionally, oncogenic signals like KRAS, BRAF, and MYC activate NRF2 transcription. 13

The signaling pathway of NRF2 is tightly regulated by *KEAP1* , which acts as a substrate adapter protein for the E3 ubiquitin ligase complex CUL3/RBX1 consisting of human cullin-3 and human RING box protein 1. 14 Extensive research has demonstrated that the KEAP1-NRF2 pathway exhibits bidirectional regulatory effects in carcinogenesis. On one hand, it possesses tumor preventive properties, whereas on the other hand, it can promote tumor progression. 15 Mutations in *NFE2L2/KEAP1* have been linked to increased tumor mutational burden (TMB) and PD-L1 expression, resulting in improved clinical responses to immunotherapy and favorable patient outcomes. 16 Additionally, lung adenocarcinoma patients with co-occurring mutations in *NFE2L2* and *KEAP1* have shown poorer survival outcomes compared with those with a single mutation in either gene. 17

The *PIK3CA* gene, located on the q26 region of the long arm of chromosome 3 (3q26), frequently undergoes activating mutations or copy number amplifications in lung cancer. 18 19 20 21 *PIK3CA* is responsible for regulating the phosphatidylinositol 3-kinase (PI3K)/Akt signaling pathway, crucial in governing cell proliferation, adhesion, differentiation, and motility. 22 Activated PI3K signaling leads to increased NRF2 accumulation in the nucleus, 23 thereby enhancing various biological processes, including de novo purine nucleotide synthesis, glutamine metabolism, and the pentose phosphate pathway. The PI3K inhibitor NVP-BKM120 reduces NRF2 expression in squamous lung cancer cells. 24 Diosmetin selectively induces apoptosis and enhances paclitaxel efficacy in NSCLC cells by accumulating reactive oxygen species through disrupting the PI3K/Akt/GSK-3β/Nrf2 pathway. 25 Recent studies have revealed the involvement of NFE2L2 in DNA repair. There exists a significant association between *NFE2L2* mutations and *ATR* gene expression. 26 NRF2 interacts with ATR at DNA damage sites, promoting *ATR* activation through its AAD-like domain and thereby facilitating G2 cell cycle arrest. 27 Notably, the *ATR* gene, a key regulator of the DNA damage response (DDR), is also located on 3q26.

Indeed, the genetic fitness of tumors is influenced by the nonadditive contributions of multiple genes within cancer pathways, underscoring the importance of interactions between mutations that may signify genetic epistasis. 28 In the context of lung cancer, the concept of epistatic mutation interactions has garnered substantial support. For example, in the realm of targeted therapy, the presence of *TP53* mutations has been linked to diminished responsiveness to tyrosine kinase inhibitors and a poorer prognosis in patients with *EGFR* -mutated NSCLC. 29 Additionally, early studies have demonstrated the mutually exclusive nature of *EGFR* and *KRAS* mutations, delineating two subtypes of NSCLC patients with distinct clinical outcomes. 30 31 Regarding immunotherapy, patients harboring concurrent *TP53* and *KRAS* mutations may potentially derive greater benefits from PD-L1 inhibitors compared with those with a single mutation. 32 33 Furthermore, the presence of *STK11* / *LKB1* mutations has been shown to facilitate resistance to PD-1/PD-L1 inhibitors in *KRAS* -mutant lung adenocarcinoma (LUAD). 34

In this comprehensive investigation, we employed targeted next-generation sequencing analysis on a robust cohort comprising 1,103 individuals diagnosed with lung cancer. Our principal objective centered on the meticulous exploration of potential deleterious co-occurrences associated with the *NFE2L2* gene within the context of lung cancer.

## Patients and Methods

### Patients and Specimens

We collected blood and tumor tissue specimens from a cohort of 1,103 individuals diagnosed with lung cancer. These patients received treatment at multiple clinical centers from January 2020 to July 2022. Prior to specimen collection, all participants provided written informed consent. The study protocol was approved by the Ethics Committee of the First Hospital of Hebei Medical University, ensuring compliance with ethical guidelines for research involving human subjects. During the selection process, patients with histopathological evidence of either lung adenocarcinoma (LUAD) or lung squamous cell carcinoma (LUSC) and who underwent standard treatment were included. Patients with other types of cancers showing multiple malignant tumor cell components (such as adenosquamous carcinoma) were excluded.

### Data Collection

High-quality total DNA was extracted from tissues using a commercial Universal Columnar Genome Extraction Kit (Kangwei, China). Sample Purification Beads (Illumina) were employed to purify fragmented DNA. To generate DNA fragments of 180 to 280 bp, hydrodynamic shearing was conducted using the M220 Focused-ultrasonicator (Covaris) on 0.6 g of genomic DNA. Subsequently, adapter-ligated libraries were generated using the TruSeq Nano DNA Sample Prep Kits (Illumina). For target enrichment, the constructed libraries were hybridized to custom-designed biotinylated oligonucleotide probes (Roche NimbleGen) covering 364 cancer-related genes ( Supplementary Table S1 , available in online version only). Subsequently, the index-coded library samples were clustered on an Illumina cBot Cluster Generation System, and the DNA libraries were sequenced using an Illumina HiSeq 2000 system. Genomic alterations, including single-nucleotide variants, small insertions and deletions (Indels), copy number alterations, and gene fusions/rearrangements, were detected with GATK, MuTect (version 1.1.4) and BreakDancer, respectively. For quality control, tumor tissue somatic mutations were refined using the following criteria: (1) variants with a frequency of <1% in the 1000 Genomes Project ( https://www.internationalgenome.org/ ) and the Exome Aggregation Consortium; (2) not present in paired germline DNA from peripheral blood lymphocytes; and (3) detected in five or more high-quality reads and without paired-end reads bias.

Data related to the TCGA cohorts were downloaded from cBioPortal ( http://cbioportal.org ). In addition, the data access period was June 2022. Briefly, we collected data on patients with lung cancer from this online database, including molecular characteristics (somatic mutations, copy number variation [CNV], and TMB), clinical information (gender, age, pathology, and smoking history), and overall survival (OS).

### Statistical Analysis

The Kaplan–Meier curves were used to estimate OS, and statistical significance was calculated using the log-rank test. Multivariate Cox analysis was used to examine the association between OS and genomic features, as well as clinical phenotypes. The related estimates were reported as hazard ratio and 95% confidence interval. For all the analysis, a *p* -value below 0.05 was considered significant. Statistical analyses were carried out using R software.

## Results

### The Prevalence and Distribution of *NFE2L2* Mutations with Lung Cancer

**Fig. 1:**
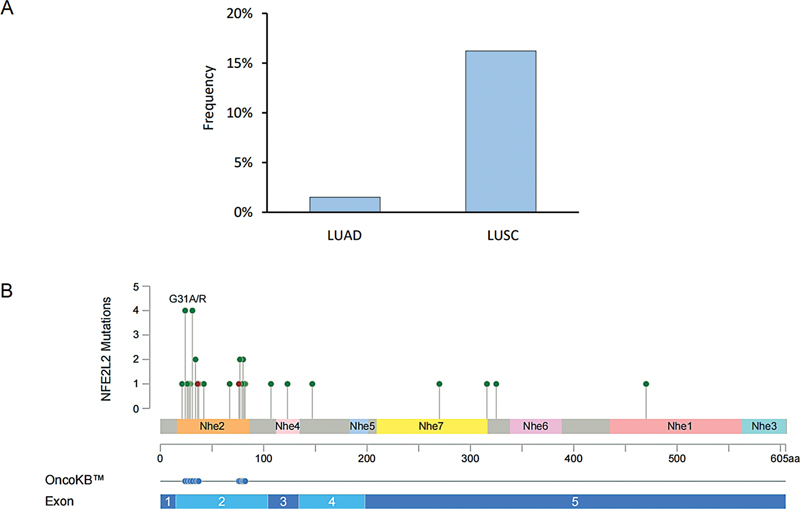
The prevalence and distribution of *NFE2L2* mutations in Chinese NSCLC. ( **A** ) The prevalence of *NFE2L2* mutations in patients with LUAD and LUSC. ( **B** ) The distribution of *NFE2L2* mutations are shown on protein schematics. Symbols indicate the mutation type and location. LUAD, lung adenocarcinoma; LUSC, lung squamous cell carcinoma; NSCLC, nonsmall cell lung carcinoma.

We conducted a retrospective analysis of sequencing data from 1,103 lung cancer patients between 2020 and 2022. The clinical characteristics of these patients were listed in Table 1 . Among the cohort of patients, a total of 33 individuals with *NFE2L2* mutations were identified. Notably, all of these individuals were male, with a median age of 66 years (range: 38–87) ( Supplementary Table S2 , available in online version only). In comparison to patients with wild-type NFE2L2, those carrying *NFE2L2* mutations exhibited a slightly higher average age ( *p* = 0.029). The prevalence of *NFE2L2* mutations is summarized in Fig. 1A , indicating a relatively high mutation frequency of 16% in LUSC. Unexpectedly, a lower mutation frequency of 3% was observed in LUAD, which is still relatively high when compared with previous reports. 16 35 Furthermore, *NFE2L2* mutations showed no significant correlation with disease stage (I/II vs. III/IV, *p* = 0.681), primary lesion location (left lung vs. right lung, *p* = 0.944), or smoking history (ever vs. never, *p* = 0.164). Aberrations in *NFE2L2* commonly arise from somatic mutations or CNVs. In our study, we identified a total of 34 *NFE2L2* variations, with 27 located in exon 2, 4 in exon 5, 2 in exon 3, and 1 in exon 4 ( Fig. 1B ). Most of them occur in DLG or ETGE motifs, and the majority of them lead to the activation of the NRF2 pathway in cancer. 36 Additionally, we identified three cases of copy number amplification variant of *NFE2L2* in LUSC, which also resulted in activation of the NRF2 pathway. Our investigation further revealed the presence of several previously unreported mutations within exon 2 of the *NFE2L2* gene. These novel mutations include D21H, V36_E45del, F37_E45del, R42P, E67Q, and L76_E78delinsQ.

**Table 1:** Clinical characteristics of nonsmall cell lung carcinoma patients with *NFE2L2* mutations and *NFE2L2* wild type

Patient characteristics	*NFE2L2* mutated ( *N* = 33)	*NFE2L2* wild-type ( *N* = 1,070)
Age (mean ± SD)	65.97 ± 9.61	61.93 ± 10.51
Gender		
Male	33	560
Female	0	510
Type		
LUSC	18	93
LUAD	15	977
Smoking status		
Ever	14	316
Never	19	752
NA	0	2
Stage		
I	9	393
II	9	227
III	14	287
IV	1	132
NA	0	31
Site		
Left lung	13	444
Right lung	20	624
NA	0	2

Abbreviations: LUAD, lung adenocarcinoma; LUSC, lung squamous cell carcinoma; NA, not applicable; SD, standard deviation.

### Identification of *NFE2L2* Co-occurring Mutations

**Fig. 2:**
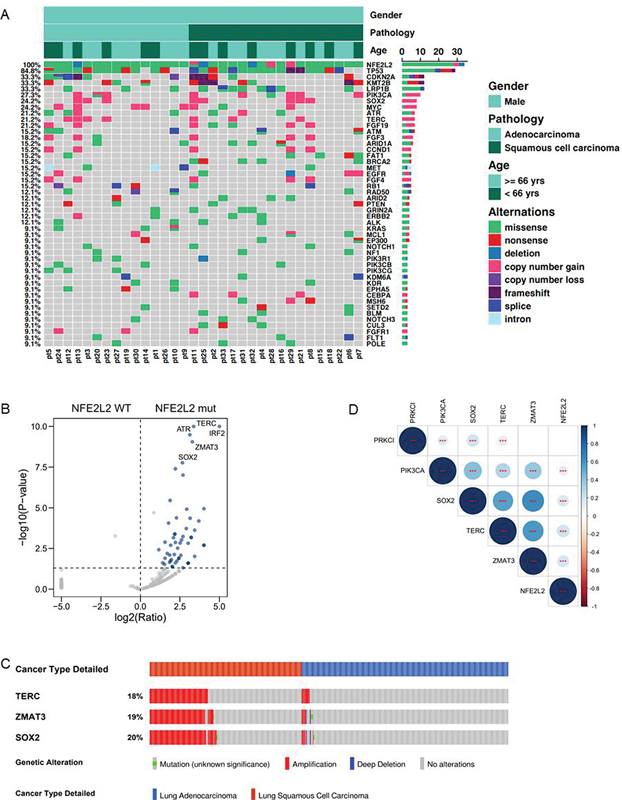
Characteristics of co-occurring gene mutations with *NFE2L2* . ( **A** ) Co-mutation genes of *NFE2L2* in patients with NSCLC. ( **B** ) Volcano plot indicating key co-occurring and mutually exclusive alterations associated with *NFE2L2* status in the Chinese NSCLC. ( **C** ) Co-occurrence pattern of *TERC* , *ZMAT3* , and *SOX2* in the TCGA cohort. ( **D** ) Heatmap illustrating the co-occurrence pattern of *NFE2L2* and genes located at chromosome 3q26. * *p* < 0.1, ** *p* < 0.05, *** *p* < 0.01. NSCLC, nonsmall cell lung carcinoma; TCGA, The Cancer Genome Atlas.

Based on the genomic data obtained from the cohort of 33 patients, we further elucidated the comprehensive landscape of *NFE2L2* gene alterations ( Fig. 2A ). Our findings indicated that individuals harboring *NFE2L2* mutations also exhibited other actionable or driver mutations, with *TP53* being the most frequently mutated gene (84.8%), followed by *CDKN2A* (33.3%), *KMT2B* (33.3%), *LRP1B* (33.3%), and *PIK3CA* (27.3%). Subsequently, a set of genes whose alterations showed the most pronounced co-occurrence with *NFE2L2* were identified ( Fig. 2B ). This gene set ( *IRF2* , *TERC* , *ATR* , *ZMAT3* , and *SOX2* ) exhibited significant co-occurrence with *NFE2L2* ( *p* < 0.001). Remarkably, a majority of these genes are situated in the q26 region of the long arm of chromosome 3 (3q26), which is frequently amplified in TCGA LUSC cohort ( Fig. 2C ). Furthermore, our study revealed that the amplification of four genes ( *PIK3CA* , *SOX2* , *TERC* , *ZMAT3* ) located on chromosome 3q26 was also found to co-occur with *NFE2L2* ( Fig. 2D ). Previous studies have suggested that *NFE2L2* is a poor prognosis marker in lung cancer. 8 37 Thus, we further investigated whether the co-occurrence of *NFE2L2* and 3q26 amplification defines a molecular subset of lung cancer with unique clinical outcomes.

### Overall Survival Features of Lung Cancer Carrying Co-mutations

**Fig. 3:**
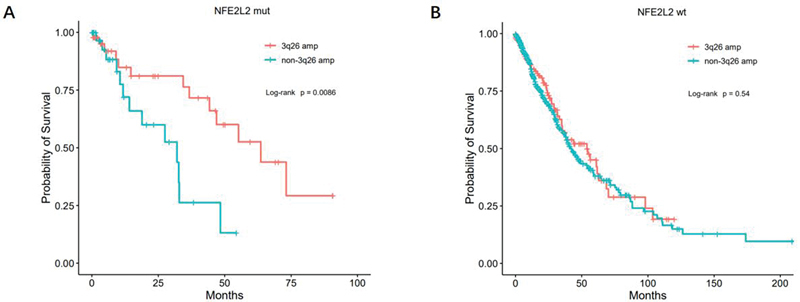
Survival analyses in the TCGA cohort. ( **A** ) Comparison of overall survival between chromosome 3q26 amplification (3q26 amp) versus non-3q26 amplification (non-3q26 amp) tumors in *NFE2L2* mutated ( *NFE2L2* mut) subcohort. ( **B** ) Comparison of overall survival between chromosome 3q26 amplification (3q26 amp) versus non-3q26 amplification (non-3q26 amp) tumors in *NFE2L2* wild-type ( *NFE2L2* wt) subcohort. TCGA, The Cancer Genome Atlas.

In order to determine whether the co-occurrence of *NFE2L2* mutations and 3q26 amplification has a different impact on the prognosis of lung cancer patients than what has been previously reported, we conducted an analysis of the TCGA Pan-Lung Cancer database ( http://cbioportal.org/msk-impact). 23 This database includes data from 1,144 patients with various types of lung cancer. After excluding patients without survival data, we ultimately analyzed 982 patients to assess survival. Our analysis revealed that in the cohort of patients with *NFE2L2* mutations, those who carried 3q26 amplification had significantly longer survival than those who did not carry the amplification ( Fig. 3A , median overall survival: 55.23 vs. 32.04, *p* = 0.0166). However, within the subgroup of patients lacking *NFE2L2* mutations, our analysis did not identify any noteworthy disparity in survival outcomes between individuals with and without 3q26 amplification ( Fig. 3B ).

### Risk of Death of Lung Cancer Carrying Co-mutations

**Fig. 4:**
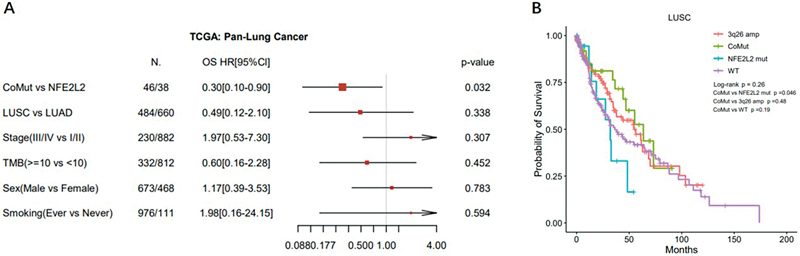
Multivariate Cox regression analysis of overall survival (OS). ( **A** ) Multivariate Cox regression analysis for OS in the TCGA Pan-Lung Cancer cohort. ( **B** ) Comparison of OS in the TCGA LUSC subcohort, including coexisting mutations (CoMut), chromosome 3q26 amplification only (3q26 amp), *NFE2L2* mutation only ( *NFE2L2* mut), and both wild-type (WT) cases. LUSC, lung squamous cell carcinoma; TCGA, The Cancer Genome Atlas.

Given the variable prevalence of *NFE2L2* mutations in LUSC and LUAD, it is essential to investigate whether the difference in prognosis associated with the presence or absence of 3q26 amplification in *NFE2L2* -mutated patients is caused by distinct clinical features. Multivariate Cox regression analyses were conducted to identify potential predictors of survival in the TCGA Pan-Lung Cancer cohort. Among the molecular characteristics and clinical information examined, the presence or absence of *NFE2L2* mutations co-occurring with 3q26 amplification was the only significant predictor of OS ( Fig. 4A ). In addition, considering that previous studies have mostly reported *NFE2L2* as a marker of poor prognosis in pan-cancer or LUAD, 8 37 38 we separately analyzed OS in TCGA LUSC patients with only *NFE2L2* mutations versus those with coexisting mutations. Our findings suggest that the synergistic effect of 3q26 amplification on *NFE2L2* still holds true, as patients carrying coexisting mutations (CoMut) had better prognoses than those with only *NFE2L2* mutations ( Fig. 4B ). These results confirmed that the co-occurrence of *NFE2L2* and 3q26 defined a molecular subset of lung cancer with better clinical outcomes compared with *NFE2L2* alone, and this difference is not related to clinical features.

## Discussion

*NFE2L2* mutations commonly coincide with additional driver mutations across diverse cancer types, such as LUSC, head and neck cancer, bladder cancer, and esophageal cancer. Consequently, the inhibition of the NRF2 pathway may exert an epistatic interaction with other driving variants, resulting in the suppression of cancer growth. 39 The primary objective of our investigation was to examine *NFE2L2* , given its high frequency of genetic alterations in lung cancer and its established correlation with adverse clinical outcomes. Furthermore, *NFE2L2* -mutated cancers often display concurrent alterations, and ongoing clinical trials (NCT05275673, NCT04518137) are evaluating *NFE2L2* as a potential therapeutic target. This offers a unique opportunity to identify key prognostic markers and potential targets for future clinical interventions. Furthermore, our investigation unveiled a significant co-occurrence of *NFE2L2* with the amplification of four genes ( *PIK3CA* , *SOX2* , *TERC* , *ZMAT3* ) located on chromosome 3q26, as indicated by the examination of co-mutation correlation patterns.

CNVs represent recurrent genetic alterations commonly identified in human tumors. The amplification of the long arm of chromosome 3 (3q) was initially documented nearly two decades ago in head and neck squamous cell carcinoma (HNSCC). 40 Subsequent evidence has demonstrated that 3q26 amplification is a frequent occurrence in malignant tumors and is potentially associated with tumor invasiveness. This suggests that 3q26 amplification plays a substantial functional role in the transition from premalignancy to malignancy in several tumor types, including laryngeal squamous cell carcinoma, HNSCC, and cervical cancer. These tumor types have been consistently observed to exhibit 3q26 amplification. 41 42 43

The SOX2 protein is a transcription factor that plays a crucial role in regulating the pluripotency of embryonic stem cells as well as in the morphogenesis and homoeostasis of tracheobronchial epithelia. The current hypothesis suggests that SOX2 is involved in various stages of invasive carcinoma development from normal epithelium, driving the expression of squamous histology markers such as P63. Studies have demonstrated that silencing SOX2 expression results in apoptosis, reduced tumorigenicity, and decreased stemness in lung cancer cells. 44 45 46 Amplification of the *SOX2* gene has been identified as a driver of its expression in various SCCs, such as lung and esophageal cancers. 47 48 Researchers have demonstrated a correlation between heightened levels of SOX2 expression and an unfavorable prognosis in both lung adenocarcinoma 49 and small cell lung cancer. 46 Paradoxically, in NSCLC patients, SOX2 expression has been linked to better clinical outcomes, indicating that complex multigenic interactions are involved in driving aggressive behavior and unfavorable clinical outcomes in tumors with 3q26 amplification. 50

Another gene that has robust evidence supporting its driving role in 3q26 amplification tumors and other tumor types is *PIK3CA* . It is located on the 3q26 genomic region, downstream of *SOX2* , and encodes the p110α protein, which is the catalytic subunit of PI3K. *PIK3CA* is responsible for regulating the PI3K/Akt signaling pathway, which is critical for cell survival in human cancer. 22 Notably, there is a higher prevalence of genetic alterations in *PIK3CA* in LUSC compared with LUAD. 18 19 Okudela and colleagues demonstrated *PIK3CA* copy number gains by FISH in 43% of Japanese LUSC patients. Similarly, Ji and coworkers noted amplification by PCR in 42% of Chinese LUSC patients. 20 21 Furthermore, a study conducted by Best et al elucidated the synergistic interplay between the KEAP1/NRF2 and PI3K pathways, which contributes to the development of NSCLC with an altered immune microenvironment. 51 The researchers observed that NRF2 exhibits oncogenic activity downstream of the PI3K pathway. Intriguingly, they also discovered that prolonged activation of NRF2 under homeostatic conditions does not trigger the development of malignant pathologies. Moreover, our investigation revealed a notable co-occurrence between *NFE2L2* and the amplification of *PIK3CA* , which is located on chromosome 3q26. However, further large-scale follow-up studies are warranted to determine whether this co-mutation has an impact on the prognosis of lung cancer patients.

*ATR* serves as a key regulator of the DDR in mammary cells, exerting a master control over this process. In cells experiencing DNA double-strand breaks, crosslinks, or replication stress, the replication protein A (RPA) envelops the single-stranded DNA (ssDNA) present at the sites of DNA damage. *ATR* effectively detects and recognizes this ssDNA coated with RPA through its interaction with the protein ATRIP. 52 Recruiting ATR/ATRIP to RPA-coated ssDNA alone is insufficient to achieve optimal activation; additional proteins are required as activators. NRF2 has emerged as a potential activator of *ATR* , playing a crucial role in maintaining genomic stability by facilitating *ATR* activation and promoting G2 cell cycle arrest. 27 We found that alterations of *ATR* also showed significant co-occurrence with *NFE2L2* . Further investigation is required to describe whether the abnormal activation of *NFE2L2* can compensate for the impairment of homologous recombination repair caused by *ATR* deficiency and effectively preserve genome stability.

*TERC* encodes the human telomerase RNA, and its increased gene expression is frequently detected in various human cancers. 53 The expression of *TERC* was differentially regulated during the oncogenesis process in the histological subtypes of lung carcinoma, with higher *TERC* expression observed in LUSC. 54 Recent studies have reported a positive feedback regulation between *TERC* and the PI3K/Akt pathway, operating independently of telomerase activity in human fibroblasts to control cell proliferation. 55 The amplification of *TERC* and its co-occurrence with *NFE2L2* in our study suggests a potential mutual synergistic effect between *TERC* and *NFE2L2* through the PI3K/Akt pathways. *ZMAT3* (Zinc Finger Matrin 3), encoding a zinc finger RNA-binding protein, is a crucial downstream tumor suppressor of the tumor protein p53. Its expression is highly dependent on p53 in *KRAS* ^G12D^ -driven LUAD, and similar to p53, *ZMAT3* inhibits LUAD growth by impeding proliferation without inducing apoptosis. 56 Interestingly, we observed that *ZMAT3* amplification is associated with a better prognosis in patients with *NFE2L2* mutations. However, no such difference was observed in patients with wild-type *NFE2L2* . We speculate that *ZMAT3* may play a positive tumor-suppressive role in the progression of *NFE2L2* -mutated tumors. Nevertheless, these speculations need validation in future in vitro or in vivo studies.

A notable observation in our study is that all 33 patients with *NFE2L2* mutations were male. Consistent with previous investigations, where among 262 patients, all 6 individuals with *NFE2L2* mutations were also male. 35 In this study, all 6 patients were smokers with LUSC. Prolonged and repetitive exposure of the respiratory tract to cigarette smoke typically triggers the activation of cellular defense mechanisms, while the substances deposited induce a multifaceted adaptive response aimed at restoring tissue homeostasis. A previous study suggests that the activation of the transcription factor NRF2 is considered a prominent characteristic of this defense system, acting as the master regulator of the cellular antioxidant response. 57 Besides *NFE2L2* 's involvement in antioxidant metabolism related to smoking, the precise mechanism underlying the higher propensity for *NFE2L2* mutations in males remains currently unclear.

This study reveals a notable disparity in the frequency of *NFE2L2* gene alterations between lung adenocarcinoma and SCC, offering crucial insights into the molecular characteristics of distinct lung cancer subtypes. This contributes to a better comprehension of the molecular classification of lung cancer, providing valuable guidance for personalized therapeutic approaches. In the TCGA Pulmonary Squamous Carcinoma project, patients harboring *NFE2L2* mutations along with 3q26 amplification exhibit prolonged median survival and superior OS. This preliminary evidence underscores the potential prognostic value of *NFE2L2* mutations in assessing the outcomes of lung cancer patients. For individuals concurrently carrying *NFE2L2* mutations and 3q26 amplification, further exploration of the prospective clinical applications within this subset is warranted. This may involve the development of more personalized treatment strategies tailored to the unique characteristics of this subgroup. In summary, this study not only sheds light on the role of *NFE2L2* mutations in lung cancer but also provides a novel perspective and insights into their potential therapeutic applications. Our study has several limitations that need to be acknowledged. Firstly, the compared subgroups had different sizes, which could introduce a potential bias in the analysis. Additionally, the lack of follow-up data limited our ability to assess long-term outcomes. To address these limitations, we utilized the TCGA survival database to complement our analysis and examine the association between genetic mutations and prognosis. However, it is important to note that discrepancies between our dataset and the TCGA database could introduce bias and restrict the generalizability of our findings to the Chinese population. Furthermore, due to the low occurrence rate of the co-occurrence in LUAD, our study may have had insufficient statistical power to fully evaluate the potential impact of this co-occurrence on prognosis in LUAD patients.

## Conclusions

Our findings demonstrate that the co-occurrence of *NFE2L2* and 3q26 is observed in approximately 3% of NSCLC cases. Notably, patients with *NFE2L2* /3q26 mutations show a more favorable prognosis compared with those with sole *NFE2L2* mutations without 3q26 amplification. As a result, further investigations should focus on elucidating whether patients with *NFE2L2* mutations may benefit from more aggressive upfront therapy when compared with individuals harboring *NFE2L2* /3q26 mutations.
