# Supplementary material for: Prevalence and Associations of Co-occurrence of NFE2L2 Mutations and Chromosome 3q26 Amplification in Lung Cancer
Source: Glob Med Genet. 2024 Apr 15;11(2):150–8. doi: 10.1055/s-0044-1786004 (PMC11018393; doi:10.1055/s-0044-1786004)
Supplement: Supplementary file 1 — Supplementary Material [file 10-1055-s-0044-1786004-s2400018.pdf]

Supplementary Table S1 List of genes in the 364 panel

364 genes covers the coding exons
ABCB1, ABL2, ACVR1B, AKT1, AKT2, AKT3, ALK, APC, AR, ARAF, ARID1A, ARID1B, ARID2, ASXL1, ATM, ATR, ATRX, AURKA, AURKB, AXIN1, AXL, BAP1, BARD1, BCL2, BCL2L1, BCL6, BCOR, BCORL1, BCR, BLM, BRAF, BRCA1, BRCA2, BRD4, BRIP1, BTK, C8orf34, CALR, CARD11, CASP8, CBFB, CBL, CCND1, CCND2, CCND3, CCNE1, CD22, CD274, CD74, CDA, CDC73, CDH1, CDK12, CDK4, CDK6, CDK8, CDKN1A, CDKN1B, CDKN2A, CDKN2B, CDKN2C, CEBPA, CHD4, CHEK1, CHEK2, CIC, CREBBP, CRKL, CRLF2, CSF1R, CSF3R, CTCF, CTNNA1, CTNNB1, CUL3, CXCR4, CYLD, CYP19A1, CYP1B1, CYP2C8, CYP2D6, DAXX, DDR1, DDR2, DICER1, DNMT3A, DOT1L, DPYD, EGFR, EP300, EPHA3, EPHA5, EPHA7, EPHB1, EPHB4, ERBB2, ERBB3, ERBB4, ERCC1, ERCC2, ERCC4, ERG, ERRFI1, ESR1, ETV5, ETV6, EWSR1, EZH2, EZR, FAM46C, FANCA, FANCC, FANCD2, FANCE, FANCF, FANCG, FANCL, FAS, FAT1, FBXW7, FGF10, FGF12, FGF14, FGF19, FGF23, FGF3, FGF4, FGF6, FGFR1, FGFR2, FGFR3, FGFR4, FH, FLCN, FLT1, FLT3, FLT4, FOXL2, FOXP1, FUBP1, GABRA6, GATA1, GATA2, GATA3, GLI1, GNA11, GNA13, GNAQ, GNAS, GRIN2A, GRM3, GSTP1, H3F3A, HDAC1, HGF, HLA-A, HLA-B, HLA-C, HNF1A, HRAS, HSD3B1, HSP90AA1, ID3, IDH1, IDH2, IGF1R, IGF2, IKBKE, IKZF1, IL7R, INHBA, INPP4B, IRF2, IRF4, IRS2, JAK1, JAK2, JAK3, JUN, KAT6A, KDM5A, KDM5C, KDM6A, KDR, KEAP1, KEL, KIT, KLHL6, KMT2A, KMT2B, KMT2C, KRAS, LRP1B, LYN, MAF, MAGI2, MAP2K1, MAP2K2, MAP2K4, MAP3K1, MAP3K13, MAPK1, MCL1, MDM2, MDM4, MED12, MEF2B, MEN1, MET, MITF, MLH1, MPL, MSH2, MSH3, MSH6, MST1R, MTHFR, mTOR, MTRR, MUTYH, MYC, MYCN, MYD88, NBN, NF1, NF2, NFE2L2, NFKBIA, NKX2-1, NOTCH1, NOTCH2, NOTCH3, NOTCH4, NPM1, NQO1, NRAS, NSD1, NTRK1, NTRK2, NTRK3, NUP93, P2RY8, PAK3, PALB2, PARP1, PAX5, PBRM1, PDCD1, PDCD1LG2, PDGFB, PDGFRA, PDGFRB, PDK1, PIK3C2B, PIK3C2G, PIK3CA, PIK3CB, PIK3CG, PIK3R1, PIK3R2, PIM1, PLCG2, PMS2, POLD1, POLE, PPARG, PPP2R1A, PRDM1, PREX2, PRKAR1A, PRKCI, PRKDC, PTCH1, PTEN, PTPN11, PTPRD, PTPRO, RAC1, RAD21, RAD50, RAD51, RAD51B, RAD51C, RAD51D, RAD52, RAD54L, RAF1, RANBP2, RARA, RB1, RBM10, RECQL, RECQL4, REL, RET, RICTOR, RNF43, ROS1, RPTOR, RRM1, RSPO2, RUNX1, RUNX1T1, SDC4, SDHA, SDHB, SDHC, SDHD, SETD2, SF3B1, SGK1, SLC34A2, SLIT2, SLX4, SMAD2, SMAD3, SMAD4, SMARCA4, SMARCB1, SMO, SNCAIP, SOCS1, SOD2, SOX10, SOX2, SOX9, SPEN, SPOP, SPTA1, SRC, STAG2, STAT3, STK11, SUFU, SYK, TAF1, TBX3, TERC, TERT, TET2, TGFBR2, TMPRSS2, TNFAIP3, TNFRSF14, TOP1, TOP2A, TP53, TSC1, TSC2, TSHR, TYMS, U2AF1, UGT1A1, VHL, WT1, XIAP, XPC, XPO1, XRCC1, XRCC2, XRCC3, ZFHX3, ZMAT3, ZNF217
31 genes covers the noncoding introns
ALK, BRAF, BRCA1, BRD4, CD74, EGFR, EML4, ERG, ETV6, EZR, FGFR1, FGFR2, FGFR3, KIT, MET, MSH2, MYC, NOTCH2, NTRK1, NTRK2, NTRK3, PDGFRA, RAF1, RET, ROS1, RSPO2, SDC4, SLC34A2, TERT, TMPRSS2, PMS2

Supplementary Table S2 Clinical characteristics and gene alterations in the 33 patients carrying *NFE2L2* mutations

Patient	Sex	Age	Pathology	Alterations
Pt1	Male	58	Adenocarcinoma	SMARCA4(c.766G > T), TP53(c.844C > T), SF3B1(c.1885G > T), **NFE2L2 (c.241G > T)**, GRIN2A(c.3793C > A), ERBB2(c.1744G > T), DDR2(c.2333G > T), CTCF(c.627G > T), JUN(c.538G > T), MET(c.3028 + 681C > G)
Pt2	Male	70	Squamous cell carcinoma	PTPRD(c.752G > T), KMT2B(c.7839del), HNF1A(c.101G > T), DNMT3A (c.1234G > T), PBRM1(c.2533del), TP53(c.991C > T), INPP4B(c.931T > A), CDKN2A(c.172C > T), **NFE2L2(c.91G > C)**, ATR(c.7423G > T), CHEK1 (c.1042A > T), PTEN(c.139A > T), ZFHX3(c.620G > T), FOXL2(c.11G > T), FGF19(c.28G > T), HRAS(c.199A > C), CHEK2(c.1066del)
Pt3	Male	66	Adenocarcinoma	RANBP2(c.2144G > C), **NFE2L2(c.76C > G)**, MET(c.1255G > A), KMT2C (c.2649A > T), CDKN2A(c.159G > C), CDKN2A(c.156G > A), NOTCH1 (c.4379A > T), TP53(c.855_856delinsTT), TERC(cnv gain), SOX2(cnv gain)
Pt4	Male	67	Squamous cell carcinoma	NF2(c.655G > A), SDHC(c.185C > G), TP53(c.713G > A), SETD2(c.5041G > T), LRP1B(c.11945A > G), FBXW7(c.1761del), PIK3C2B(c.4619C > G), PMS2(c.-87-u114C > T), ATM(c.8010 + 1G > T), IRF2(c.571G > C), BLM (c.2333C > G), CUL3(c.1351G > A), ERBB2(c.929C > T), CDC73(c.803G > A), SPOP(c.337G > A), SPOP(c.229G > A), PTPN11(c.1303G > A), ALK(c.2730G > C), BRCA2(c.9065G > A), **NFE2L2(c.199G > C)**, EP300(c.1775T > G), BRCA2 (c.7778G > T), RICTOR(c.1094C > T), BCOR(c.1334C > T), ATM(c.2587G > A)
Pt5	Male	55	Adenocarcinoma	ROS1(c.3184G > T), CDKN2A(c.351_352delinsTC), CRLF2(c.689C > G), KMT2B(c.2479C > T), **NFE2L2(c.72G > C)**, MAGI2(c.550G > T), IKZF1 (c.1094C > T), TP53(c.859G > T), PIK3CG(c.398G > A), TP53(c.743G > C), MET(c.3028 + 164del), ATM(c.112G > A), ERBB3(c.1442G > A), ATR(c.3613C > G), CCND1(cnv gain), FGF19(cnv gain), FGF3(cnv gain), FGF4(cnv gain)
Pt6	Male	72	Squamous cell carcinoma	**NFE2L2(c.100C > G)**, SETD2(c.4283G > T), ERCC1(c.239C > T), FAT1(c.8111C > G), RAD50(c.3424G > C), TNFAIP3(c.1739A > T), CDKN2A(c.205G > T), ACVR1B(c.1154A > T), EPHA3(c.1772C > T), GNAS(c.280G > T), BLM (c.2474C > T), IL7R(c.947C > A), MSH3(c.1774G > T), KMT2B(c.7551del), FLT1(c.1661-2A > T), TERT(c.-58-u66C > T), LRP1B(c.11771_11772delinsTT), SMO(c.1261C > T), MCL1(c.901A > G), NF1(c.3545T > C), EGFR(cnv gain)

Supplementary Table S2 (*Continued*)

Patient	Sex	Age	Pathology	Alterations
Pt7	Male	38	Squamous cell carcinoma	**NFE2L2(c.85G > C)**, BRCA2(c.640G > A), ASXL1(c.991C > G), KDM6A(c.655-1G > C), DICER1(c.5332G > A), KMT2B(c.15844C > T), RPTOR (c.139C > A), TSC1(c.163C > T), MST1R(c.1021G > A), PRKDC(c.7864G > T), ARID1B(c.3248G > A), IGF1R(c.430G > C), PTEN(c.176C > G), EP300(c.5626C > T), ATM(c.2662G > C), FAT1(c.12868G > A), EP300(c.6181C > G), TOP2A (c.634G > A), MAP3K1(c.1156G > C), ATM(c.6705_6706delinsAT), EP300 (c.3955G > A), PRKAR1A(c.134G > A), PIK3CA(cnv gain), EGFR(cnv gain)
Pt8	Male	55	Squamous cell carcinoma	RB1(c.1333-2A > G), TP53(c.503A > T), KDR(c.2096T > C), RECQL(c.1208G > C), **NFE2L2(c.229G > T)**, CSF1R(c.1702C > G), CSF1R(c.1695del), MSH6 (c.71C > A), EPHB1(c.2565G > C), ROS1(c.4672C > A), MET(c.2255C > T), NOTCH1(c.4475A > G), SOX2(cnv gain), CCND1(cnv gain), FGF19(cnv gain), FGF3(cnv gain), FGF4(cnv gain)
Pt9	Male	87	Adenocarcinoma	NF2(c.1475_1500del), ARID1A(c.184G > A), NTRK1(c.326A > G), **NFE2L2 (c.235G > C)**, MET(c.3028 + 1G > T), MYC(cnv gain)
Pt10	Male	69	Adenocarcinoma	KRAS(c.34G > T), ATM(c.2922-1G > A), ALK(c.872G > T), ATR(c.1731A > T), ALK(c.4045G > A), **NFE2L2(c.320A > T)**, RAD50(c.3475G > T), EPHA5 (c.1021G > T), LRP1B(c.521G > T), RAD50(c.2923-1G > T), KDR(c.1582T > C), **NFE2L2(c.368T > C)**, CDKN2B(cnv loss), CDKN2A(cnv loss), KRAS(cnv gain)
Pt11	Male	52	Squamous cell carcinoma	TP53(c.920-2A > T), CDKN2A(c.389del), **NFE2L2(c.440T > A)**, FANCA (c.3275G > T), KMT2B(c.6116C > G), NOTCH1(c.1815C > G), RB1(c.1399C > G), BRCA2(c.7505G > T), GRIN2A(c.748G > A), SLX4(c.2899G > A), MYD88 (c.204C > A), RSPO2(c.433T > A), CASP8(c.1346C > T), PREX2(c.2857T > G), CEBPA(cnv gain), TERC(cnv gain), MLH1(cnv loss), FGF3(cnv gain), FGF4(cnv gain), SOX2(cnv gain), RPTOR(cnv gain), CASP8(cnv gain), PIK3CA(cnv gain), CCND1(cnv gain), CCNE1(cnv gain), MYC(cnv gain), FGF19(cnv gain), SPOP (cnv gain), SF3B1(cnv gain), **NFE2L2(cnv gain)**, FGFR1(cnv gain)
Pt12	Male	69	Adenocarcinoma	SLX4(c.4577C > G), CDKN2A(c.151-2A > T), TP53(c.701A > G), TGFBR2 (c.1493C > T), EPHB1(c.2682C > G), PTEN(c.176C > G), LRP1B(c.2132G > C), PRDM1(c.1111C > T), ERBB4(c.2761T > C), PTPN11(c.854-2A > G), PIK3CG (c.3058C > T), EPHA5(c.2194G > T), ATR(c.1219A > G), RAD50(c.3333G > C), **NFE2L2(c.229G > C)**, FAT1(c.8445G > C), TERC(cnv gain)
Pt13	Male	62	Adenocarcinoma	**NFE2L2(c.105_134del)**, PDGFRA(c.1028C > T), CDKN2A(c.102dup), ACVR1B (c.1297A > G), TP53(c.483_485delinsGGA), ATR(cnv gain), TERC(cnv gain), PIK3CA(cnv gain), SOX2(cnv gain), MYC(cnv gain), CCND1(cnv gain), FGF19(cnv gain), FGF4(cnv gain), FGF3(cnv gain), MAP2K1(cnv gain), SMAD3(cnv gain), ERBB2(cnv gain), STAG2(cnv loss)
Pt14	Male	59	Adenocarcinoma	**NFE2L2(c.238A > G)**, SETD2(c.2216A > C), PIK3CB(c.2897A > G), TET2 (c.2570A > T), KRAS(c.34G > T), KMT2B(c.10616G > C), KMT2B(c.8704C > T), CREBBP(c.6428A > G), STK11(c.598-1G > T), EP300(c.193C > T), MYC(cnv gain)
Pt15	Male	75	Squamous cell carcinoma	FANCD2(c.1355C > A), FAT1(c.13371T > G), KAT6A(c.2515G > A), **NFE2L2 (c.238A > G)**, PARP1(c.370A > G), TP53(c.811G > A)
Pt16	Male	79	Squamous cell carcinoma	ARID1A(c.3055G > C), ARID1A(c.3094G > T), ATR(c.5814G > T), ATR (c.5059G > C), DNMT3A(c.1646G > A), EPHB4(c.1616C > T), FLT1(c.2509G > T), KDM5A(c.1406C > T), LRP1B(c.4927G > A), **NFE2L2(c.79G > C)**, **NFE2L2 (c.61G > C)**, PIK3CB(c.1264C > T), SRC(c.344A > G), TP53(c.848G > C)
Pt17	Male	81	Squamous cell carcinoma	ARID1A(c.802C > G), TP53(c.97-2A > C), CYLD(c.2296A > T), CDKN2A (c.226G > A), SMAD2(c.15G > T), **NFE2L2(c.1409T > C)**, MTOR(c.4533G > T), FGF19(c.121C > A), LRP1B(c.2333G > T), ERBB2(c.2005G > T), LRP1B (c.2338C > A), CEBPA(cnv gain), STK11(cnv loss), TERC(cnv gain)
Pt18	Male	65	Squamous cell carcinoma	TSC2(c.5202T > G), TSC2(c.1513C > T), TP53(c.1051A > T), **NFE2L2 (c.92G > C)**
Pt19	Male	76	Adenocarcinoma	**NFE2L2(c.83T > C)**, KDM6A(c.443 + 1G > T), LRP1B(c.12298T > C), TP53 (c.1024C > T), CARD11(c.352A > G), EPHA5(c.1169C > G), FGFR1(cnv gain)
Pt20	Male	69	Adenocarcinoma	TSC2(c.94G > T), MAP3K13(cnv gain), KLHL6(cnv gain), PIK3CA(cnv gain), PIK3R1(c.1841A > G), FGF23(c.468G > T), PIK3CA(c.3140A > G), **NFE2L2 (c.101G > C)**, ARID1A(c.3068G > T), FGF3(c.134G > T), EPHA7(c.1129G > C), TOP1(c.1406A > T), FLT1(c.160A > T), TP53(c.518T > G), NF1(c.1733T > C)

(*Continued*)

 © 2024. The Author(s).

Supplementary Table S2 (*Continued*)

Patient	Sex	Age	Pathology	Alterations
Pt21	Male	66	Squamous cell carcinoma	TP53(c.800del), **NFE2L2(c.92G > C)**, IKZF1(c.727G > A), SOX2(cnv gain), CD274(cnv gain), CEBPA(cnv gain), PIK3CA(cnv gain), TERC(cnv gain), PDCD1LG2(cnv gain), JAK2(cnv gain)
Pt22	Male	66	Squamous cell carcinoma	**NFE2L2(c.72G > C)**, TP53(c.920-2A > G)
Pt23	Male	65	Adenocarcinoma	AKT3(c.1271C > A), **NFE2L2(c.70T > C)**, ARID1B(c.4084C > T), KMT2B (c.11821C > T), KMT2B(c.11701C > T), KMT2B(c.11533C > T), NTRK3 (c.330C > G), TP53(c.329G > T), TERC(cnv gain), PIK3CA(cnv gain), SOX2(cnv gain)
Pt24	Male	62	Adenocarcinoma	KRAS(c.35G > T), SRC(c.1376C > G), TP53(c.461G > T), ATM(c.937T > C), FLT4(c.278G > T), ALK(c.4034G > T), **NFE2L2(c.947C > T)**, ERBB4 (c.2160_2161delinsTT), IDH1(c.395G > A), CDKN2A(c.247C > T), PIK3CB (c.2003G > A), RB1(cnv loss), CDKN2B(cnv loss), CDKN2A(cnv loss), MYC(cnv gain), EGFR(cnv gain), FGFR1(cnv gain)
Pt25	Male	57	Squamous cell carcinoma	TP53(c.746G > C), SMAD4(c.779dupA), **NFE2L2(c.227_232del)**, ZFHX3 (c.2747G > T), KMT2B(c.1295del), EGFR(c.3329A > G), ZNF217(c.1450T > A), CDKN2A(c.56_81del), LRP1B(c.7259G > T), BLM(c.1424G > T), PDCD1 (c.363C > A), CUL3(c.539G > C), BRCA2(c.7060C > T), BRD4(c.3650C > G), IRS2(c.3631C > T), IRF2(c.456T > G), SMAD3(c.77A > T), ALK(c.562C > T), PIK3R1(c.1735_1740del), SOX2(cnv gain), FOXL2(cnv gain)
Pt26	Male	74	Adenocarcinoma	FGF10(c.236G > T), FLT3(c.2699A > G), INHBA(c.539del), **NFE2L2(c.809C > T)**, **NFE2L2(c.245A > G)**, TOP1(c.1340C > T), TP53(c.592G > T)
Pt27	Male	77	Adenocarcinoma	ARID2(c.5026C > T), CARD11(c.1900G > C), CD22(c.1517G > A), GNAS (c.592C > G), KMT2B(c.6257C > T), MDM2(c.504G > T), **NFE2L2(c.125G > C)**, PIK3CG(c.1128C > A), PIK3R1(c.514G > T), POLE(c.3866G > T), PTEN(c.389G > A), PTPRO(c.3609C > G), RB1(c.1050-2A > G), SPTA1(c.916C > T), TP53(c.734G > T)
Pt28	Male	68	Squamous cell carcinoma	AKT2(c.1120A > T), ARID2(c.3048A > T), DOT1L(c.3806A > G), FANCG(cnv gain), LRP1B(c.7762 + 2T > A), **NFE2L2(cnv gain)**, PIK3CA (c.1462G > C), REL(c.1507A > T), SPTA1(c.1717A > G), SUFU(c.1156A > T), TP53(c.536A > G)
Pt29	Male	57	Squamous cell carcinoma	TP53(c.98del), FAT1(c.6923C > T), POLE(c.3823C > T), KMT2C(c.6766A > C), NOTCH4(c.5326C > T), ARID2(c.2644G > T), EGFR(c.2235_2249del), MCL1(c.932G > T), FGF3(cnv gain), FGF4(cnv gain), MSH6(cnv gain), SOX2(cnv gain), XPO1(cnv gain), PIK3CA(cnv gain), CCND1(cnv gain), MYC (cnv gain), FGF19(cnv gain), MCL1(cnv gain), **NFE2L2(cnv gain)**, BRD4(cnv gain), MSH2(MSH2-EIF2C2 fusion)
Pt30	Male	74	Adenocarcinoma	TP53(c.742C > T), RB1(c.2242G > T), **NFE2L2(c.973T > C)**, KDM5A(c.254G > T), MUTYH(c.937G > C), NOTCH3(c.6604G > A), KDR(c.454G > A), RAD50(cnv loss), DAXX(cnv gain), CDKN1A(cnv gain), MYC(cnv gain), MCL1 (cnv gain)
Pt31	Male	66	Squamous cell carcinoma	KMT2B(c.3244G > T), PTPRD(c.2630A > T), FBXW7(c.1204_1207del), **NFE2L2 (c.92G > C)**, TP53(c.730G > T), KDM6A(c.3025G > A), PDGFRB(c.2128C > A), LRP1B(c.6493C > T), GRIN2A(c.1630G > T), SMARCA4(c.2476G > T), BRCA2 (c.520C > G), APC(c.7004G > T), FLT4(c.81G > T), GRIN2A(c.721A > T), MSH6 (c.188C > G), LRP1B(c.5960G > T), NTRK1(c.1569G > T), NF1(c.6077C > T), PIK3CA(cnv gain), ATR(cnv gain)
Pt32	Male	63	Squamous cell carcinoma	**NFE2L2(c.110_136del)**, FUBP1(c.313 + 3A > C), KMT2B(c.3246G > T), KMT2B (c.5814G > T), ARID1A(c.6709G > T), JAK2(c.1132G > A), GRIN2A(c.1547A > T), TP53(c.730G > T), NOTCH3(c.4793A > G)
Pt33	Male	58	Squamous cell carcinoma	CUL3(c.1557G > A), LRP1B(c.12340 + 1G > T), ARID2(c.422A > T), LRP1B (c.13142A > T), POLE(c.4145G > A), **NFE2L2(c.70T > C)**, MYC(c.745A > T), PARP1(c.859A > G), RICTOR(c.772A > T), TP53(c.743G > A), NOTCH3 (c.5108G > T)

 © 2024. The Author(s).
